# Hot Quadrant Sign in a Lung Cancer Patient: A Rare Hepatic Pseudo-Lesion Unveiling Superior Vena Cava Obstruction

**DOI:** 10.7759/cureus.89952

**Published:** 2025-08-12

**Authors:** Muhammad Mohsin Zahoor, Abdul Mannan, Hira Gul, Ali Raza, Brian Casserly

**Affiliations:** 1 Respiratory Medicine, University Hospital Limerick, Limerick, IRL

**Keywords:** collateral venous circulation, hot quadrant sign, mediastinal mass, pseudo lesion, pulmonary adenocarcinoma, segment iv liver enhancement, superior vena cava obstruction (svco)

## Abstract

The hot quadrant sign is a rare yet clinically sensitive radiologic phenomenon characterized by focal enhancement of segment IV of the liver. It arises due to portosystemic venous shunting, most commonly due to superior vena cava obstruction (SVCO). This case report presents a 61-year-old male patient with a significant smoking history, weight loss, and symptoms suggestive of intrathoracic malignancy. Imaging confirmed a large right hilar and upper lobe pulmonary mass with extensive mediastinal invasion, complete SVCO, and a well-visualized hot quadrant sign on CT. The hepatic enhancement observed in segment IV was secondary to collateral venous flow via internal thoracic veins, a finding that could easily be mistaken for a hypervascular hepatic lesion. Early identification of this sign facilitated recognition of SVCO, supported a diagnosis of advanced lung adenocarcinoma, and prevented misinterpretation of liver imaging. This case emphasizes the clinical and educational importance of recognizing hepatic pseudo-lesions linked to systemic vascular abnormalities.

## Introduction

Superior vena cava obstruction is an uncommon but significant consequence of intrathoracic malignancy, i.e., lung adenocarcinoma, typically manifesting with venous congestion and formation of collateral pathways. One rarely encountered yet diagnostically relevant imaging feature in SVCO is the hot quadrant sign, a focal, wedge-shaped hyperattenuation in segment IV of the liver on contrast-enhanced CT. This enhancement arises due to the rerouting of venous blood from the internal thoracic and cardio-phrenic veins to the left portal vein, creating a pseudo-lesion that may be misidentified as a focal liver tumour or metastasis. The phenomenon was first described in nuclear imaging literature and later recognized on CT scans in the context of systemic venous obstruction [1]. Despite its utility in identifying SVCO, it remains infrequently reported in clinical literature, making this case a valuable contribution to diagnostic radiology and thoracic oncology.

## Case presentation

**Figure 1:**
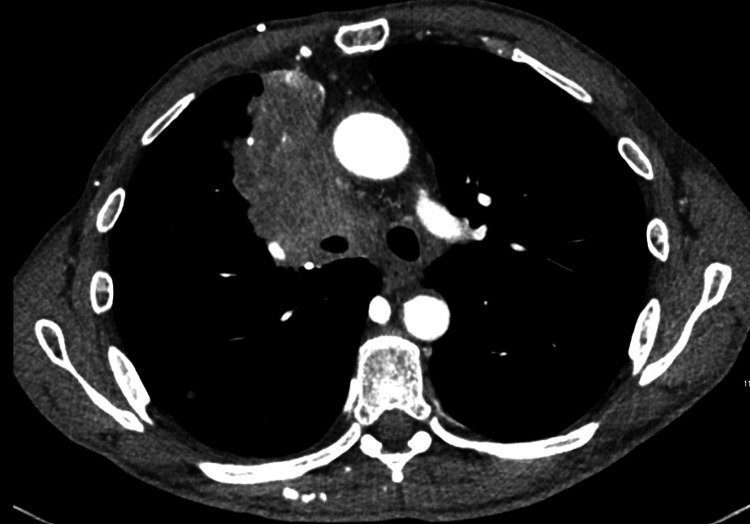
Contrast-enhanced CT thorax shows a pulmonary mass originating from the right upper lobe, with direct invasion into the mediastinum and occlusion of the superior vena cava

A 61-year-old male patient with a 35-year pack history of smoking was referred to the rapid access lung clinic with a persistent cough, shortness of breath, and documented unintentional weight loss. Physical examination was notable for facial swelling and mild venous distention in the neck and chest, raising concern for an obstructive central thoracic process. Chest radiography revealed a large right hilar mass extending superiorly. A contrast-enhanced CT thorax demonstrated a sizeable pulmonary mass originating from the right upper lobe, with direct invasion into the mediastinum and complete occlusion of the superior vena cava (Figure 1).

**Figure 2:**
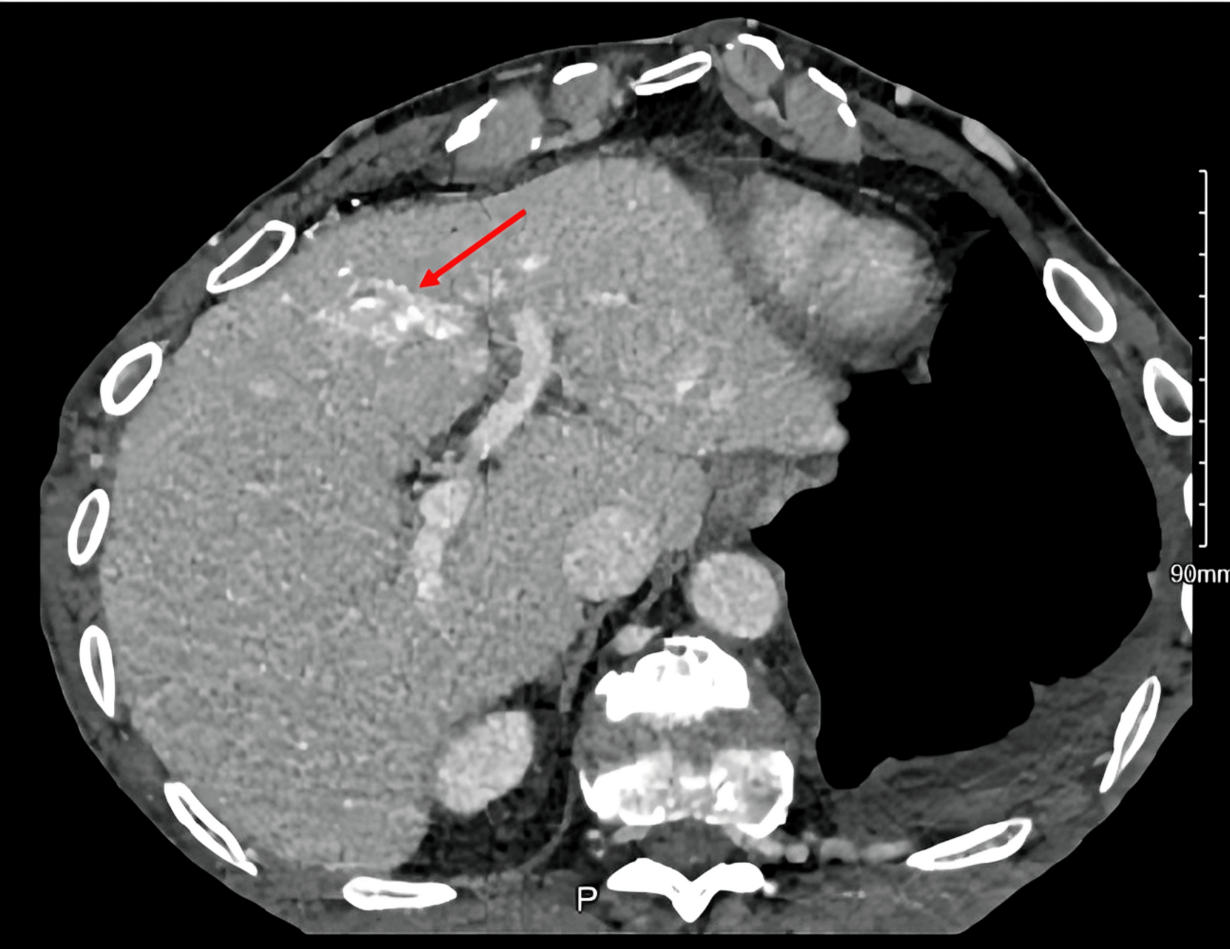
Axial view of CT abdomen showing focal area of contrast accumulation in segment IV of liver (arrows) showing hot quadrant sign.

**Figure 3:**
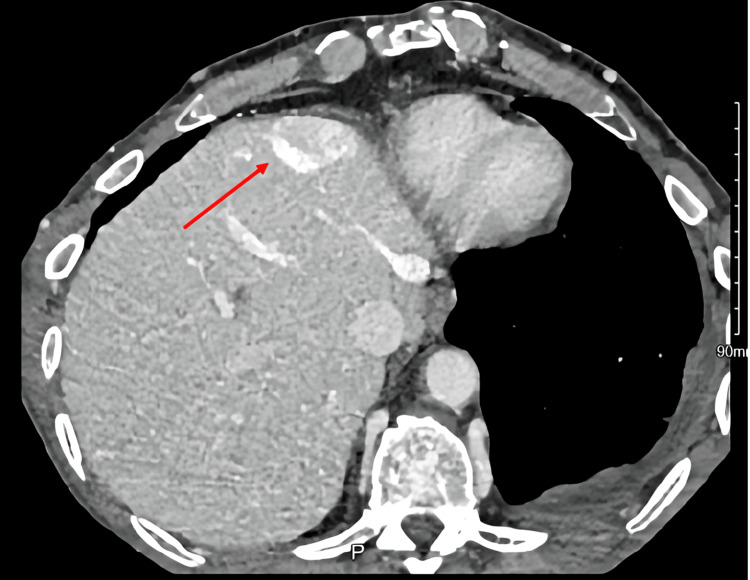
Axial view of CT abdomen showing hot quadrant sign in segment IV of liver

Extensive collateral venous circulation was visualized, including hypertrophy of the azygous system, right supra scapular vein, intercostal veins, and internal thoracic veins. Notably, there was wedge-shaped hyperattenuation in segment IV of the liver, adjacent to the falciform ligament, consistent with the hot quadrant sign (Figures 2-3).

Additional hypertrophy of the right cardio-phrenic venous vasculature further supported the diagnosis. Importantly, the IVC itself remains patent, and there is no direct evidence of IVC obstruction. The imaging findings reflect compensatory collateral circulation due to SVC obstruction, not intrinsic IVC pathology. Endobronchial ultrasound-guided biopsy of the mediastinal mass confirmed pulmonary adenocarcinoma. The patient underwent radiotherapy, resulting in a reduction in tumour size and partial relief of obstructive symptoms. Chemotherapy was subsequently started, and the patient continued oncologic management under multidisciplinary care.

## Discussion

The hot quadrant sign, although rare, is a compelling radiologic indicator of SVCO. It results from portosystemic collateral formation, a compensatory mechanism for impaired venous return to the heart. In this case, the liver’s segment IV enhancement represented redirected venous blood from the internal thoracic right cardio phrenic into the left portal vein, bypassing the obstructed SVC. The CT appearance can closely mimic hepatic neoplasms, potentially leading to misdiagnosis and unnecessary interventions. Recognition of this sign requires awareness of its vascular origin, typical location, and associated collateral circulation [1,2].

In SVCO, the hot quadrant sign is most often seen when contrast is injected into an upper limb vein during arterial or early portal phases. Although the sign has been reported in isolated cases, such as those following central venous catheterization, malignancy-related SVCO remains its most serious and clinically relevant context [2,3]. Kobayashi et al. reviewed hepatic pseudo lesions in the third inflow area, emphasizing their potential to mimic hepatocellular carcinoma and the importance of distinguishing vascular anomalies from true neoplasms [4]. Nassar et al. presented a radiologic case of SVCO with a hot quadrant sign, further validating its specificity in systemic venous obstruction and its utility in avoiding misclassification of hepatic lesions [5]. Dickson also emphasized the importance of recognizing the focal hepatic hot spot sign as a reliable indicator of SVCO in radiologic practice [6].

This case is unique because it documents the hot quadrant sign in a patient with lung adenocarcinoma, causing complete SVC obstruction, alongside extensive collateral pathways. It adds to the limited published literature and serves as a critical reminder of the systemic manifestations of thoracic malignancies. Importantly, recognizing the hepatic pseudo-lesion prevented misclassification as metastatic disease, which could have altered staging and treatment plans.

## Conclusions

The hot quadrant sign represents a rare but diagnostically important pseudo-lesion in the liver, typically indicating SVCO from an upstream pathology such as malignancy. This case highlights the need for careful interpretation of segment IV enhancement in the presence of mediastinal disease, venous collaterals, or clinical signs of SVCO. Awareness of this finding among clinicians and radiologists can expedite accurate diagnosis, optimize management, and avoid unnecessary hepatic investigations or procedures.
